# Adherence of Surgeons to Antimicrobial Prophylaxis Guidelines in a Tertiary General Hospital in a Rapidly Developing Country

**DOI:** 10.1155/2013/842593

**Published:** 2013-12-23

**Authors:** Ahmed Abdel-Aziz, Ayman El-Menyar, Hassan Al-Thani, Ahmad Zarour, Ashok Parchani, Mohammad Asim, Rasha El-Enany, Haleema Al-Tamimi, Rifat Latifi

**Affiliations:** ^1^Section of Trauma Surgery, Department of Surgery, Hamad General Hospital (HGH), P.O. Box 3050, Doha, Qatar; ^2^Clinical Research, Section of Trauma Surgery, Hamad General Hospital (HGH), P.O. Box 3050, Doha, Qatar; ^3^Clinical Medicine, Weill Cornell Medical School, P.O. Box 24144, Doha, Qatar; ^4^Department of Medicine, Ahmed Maher Teaching Hospital, Cairo, Egypt; ^5^Department of Pharmacy, Hamad General Hospital (HGH), P.O. Box 3050, Doha, Qatar; ^6^Department of Surgery, University of Arizona, P.O. Box 245005, Tucson, AZ, USA

## Abstract

*Objectives*. To assess the standard practice of care of surgeons regarding surgical antibiotic prophylaxis, to identify gaps, and to set recommendations. *Methods*. A retrospective analysis of data obtained from different surgical units in a single center in Qatar over a 3-month period in 2012. A total of 101 patients who underwent surgery and followed regimes for surgical prophylaxis as per hospital guidelines were included in the study. *Results*. The overall use of antibiotic was 89%, whereas the current practice did not match the recommended hospital protocols in 53.5% of cases. Prolonged antibiotics use (59.3%) was the commonest reason for nonadherence followed by the use of an alternative antibiotic to that recommended in the protocol (31.5%) and no prophylaxis was used in 9.2% of cases. The rate of compliance was significantly higher among clean surgery than clean contaminated group (*P* = 0.03). Forty-four percent of clean and 65% of clean-contaminated procedures showed noncompliance with the recommended surgical antimicrobial prophylaxis hospital guidelines. *Conclusion*. Lack of adherence to hospital protocols is not uncommon. This finding remains a challenge to encourage clinicians to follow hospital guidelines appropriately and to consistently apply the surgical antibiotic prophylaxis. The role of clinical pharmacist may facilitate this process across all surgical disciplines.

## 1. Introduction

**Figure 1:**
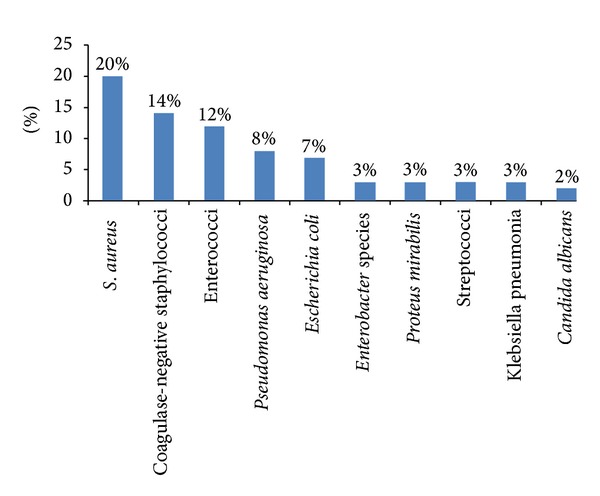
The 10 frequently identified types of pathogens responsible for surgical site infections in hospitals (adopted from [5]).

Surgical antimicrobial prophylaxis (SAP) is an initial administration of short course of an antimicrobial agent prior to surgery in order to prevent surgical site infections [1]. SAP is critical in preventing infections that may lead to sepsis, organ failure, and death during hospital stay. Despite huge advances in antiseptic measures, antibiotics, and preoperative precautions, surgical site infection (SSIs) still accounted for high morbidity and mortality [2]. SSI is the second most common type of health care-associated infection after urinary tract infections [3]. Also, SSI was reported to represent 14–16% of the estimated two-million nosocomial infections affecting hospitalized patients in the United State [4]. It has been reported that at least 5% of patients undergoing a surgical procedure developed SSI [5]. Kirkland et al. [6] showed that patients who developed SSI have 60% more chances of prolonged intensive care unit stay, five fold increased risk of readmission to the hospital and two-times higher rate of mortality compared to patients who had no SSI. One of the most common microorganisms that are involved in SSI is *Staphylococcus aureus*, which is reportedly the cause of 20% of SSI in general hospitals (Figure 1) [7].

Despite numerous factors that contribute to the risk of SSI, the increase in degree of intraoperative surgical wound contamination remains the most established risk factor [8]. Culver et al. [9] found that dirty wounds have higher rates of SSI compared to clean wounds. The odds ratio for SSI per 100 operations was 7.1 for dirty procedures and 2.1 for clean procedures. Therefore, SAP is essential to prevent SSI and its complications; thereby it helped to improve wound healing process and eventually reduce the overall hospital stay [10]. Most of the published guidelines clearly recommend discontinuation of SAP after wound closure, and many studies which compared single dose prophylaxis versus multiple doses failed to show any benefits of the multiple doses [11]. Dellinger et al. [12] observed no benefit of prolonged and inappropriate use of antibiotics during the postoperative phase and found an increased risk of nosocomial infections with resistant strains.

The SAP guidelines at Hamad General Hospital (HGH) were developed in 2006 based on international recommendations and were evolved during continuous process of modifications. Interestingly, the rate of compliance with the institutional SAP guidelines varies in the literature. However, there is lack of evidence to support the standard of care and the compliance with surgical antimicrobial prophylaxis guidelines in our organization. There is a potential opportunity for a clinical pharmacist to facilitate this process across all surgical disciplines. Herein, to maximize the health care and mitigate the gap between both practice and evidence based recommendations, we aimed to evaluate the current standard practice of care in the surgical intensive care at HGH by investigating whether the surgical antibiotic prophylaxis guidelines are correctly implemented for patients undergoing surgical procedures.

## 2. Methods

Retrospective analysis of data obtained from different surgical units at HGH was performed during a three-month period. Our HGH hospital is the main tertiary hospital in Qatar and it comprises nine operating theatres.

### 2.1. Inclusion Criteria

The study population included all patients from April to June 2011 who were scheduled for major surgery that required SAP for the clean or clean-contaminated surgery as per the current guidelines.

### 2.2. Exclusion Criteria

Contaminated surgeries were excluded because antibiotics would be routinely administered as a therapeutic intervention. Also, we excluded surgery for infants, cancer, and gynecological purposes, as well as surgery that did not imply clear regimen for prophylaxis or hospital guidelines. Operation theatre log books were reviewed for patients who underwent surgery and were administered with SAP regimen according to the hospital guidelines. Data were collected from operation theatre log books (as there is no computerized database in operation theatre), medical records, medication profile, microbiological cultures, and septic workup available in the electronic Medical Records (eMR) viewer of the hospital. Data included patients' gender, age, type of surgery, antibiotic allergy, history of chronic illness, antibiotic type, antibiotic dose, antibiotic route of administration, and duration of antibiotic use. The patients' microbiological data were confirmed for no current infection, and the antibiotics prescribed were only used for surgical prophylaxis.

### 2.3. Definitions

Clean wound is considered when the operative procedure does not enter into a normally colonized viscus or lumen of the body [13]. Clean-contaminated wounds are those in which the operative procedure enters into a colonized viscus or cavity of the body, but under elective and controlled circumstances whereas the contaminated wounds are those in which gross contamination is present at the surgical site in the absence of obvious infection [13].

### 2.4. Statistical Analysis

This is a descriptive, retrospective, observational analysis. Data were presented as proportions, mean ± standard deviation (SD) or median and range, whenever applicable. Pearson chi-square (*χ* ^2^) test was used to analyze the categorical variables. Association between different antibiotic types and compliance with hospital SAP guidelines (compliance versus noncompliance) and assessment of surgeon adherence to antibiotic prophylaxis guidelines (compliance versus noncompliance) were performed. A significant difference was considered when the *P* value was less than 0.05. Data analysis was carried out using the Statistical Package for Social Sciences version 18 (SPSS Inc., Chicago, IL). This study has been approved by the Medical Research Center at Hamad Medical Corporation, Qatar (IRB# 11226/11).

## 3. Results

**Figure 2:**
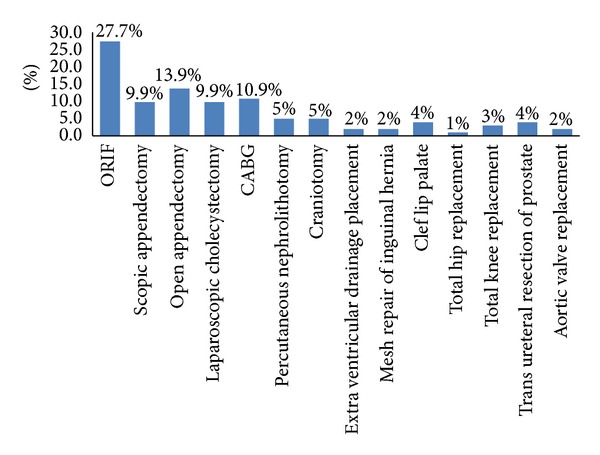
Types of surgery.

Of the total 250 patients who undergone surgery, 101 fulfilled the inclusion criteria and were included in the study. The remaining 149 patients were excluded because of lack of clear regimes for surgical prophylaxis according to hospital guidelines. The majority of patients were males (80%) with mean age of 39.9 ± 17 yrs. The study included 14 different categories of surgery for evaluation according to the hospital infectious disease SAP guidelines. The major classes of surgery were clean (54.5%) and clean contaminated (45.5%) (Table 1). Open reduction internal fixation surgery (ORIF) (27.7%) and appendectomy (13.9%) were the most frequently performed surgical procedures.  Figure 2 shows different types of surgeries involved in the study. The overall use of antibiotic was 89%, and the most commonly used antibiotics were cefazolin (44.6%), cefuroxime (17.8%), and ceftriaxone (16.8%). Contrarily, Co-amoxicliv (Amoxicillin + clavulanic acid) (5.9%), metronidazole (2%), vancomycin (1%), and ciprofloxacin (1%) were used less frequently.

**Figure 3:**
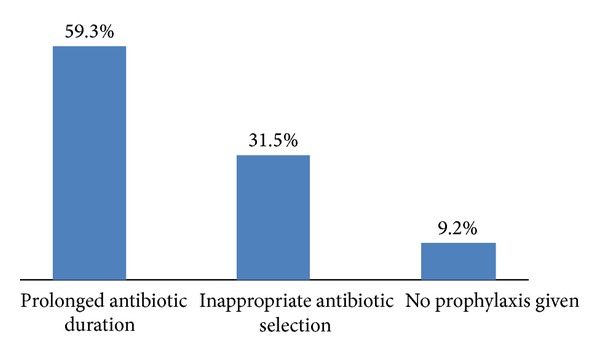
Reasons for noncompliance.

The overall rate of compliance with the hospital SAP guidelines was 46.5% and the remaining 53.5% cases did not comply (Table 1). The main reasons for noncompliance with the recommended guidelines were prolonged antibiotic duration (59.3%) and inappropriate selection of antibiotic (31.5%) for the surgery which needs prophylaxis. The remaining 9.2% cases did not receive antibiotic prophylaxis despite the clear indications as per the hospital guidelines (Figure 3). The compliance rate was significantly higher for clean surgery than clean contaminated group (66% versus 34%; *P* = 0.03). Moreover, 43.6% of the clean and 65.2% of the cleancontaminated procedures were considered noncompliant (Table 2).

Due to the small numbers of surgeries in the study, interventions were grouped according to the main surgical categories; for example, inguinal hernia, cholecystectomy, and open and laparoscopic appendectomy were grouped as general surgery, whereas craniotomy and extra ventricular drainage placement were compiled under neurosurgery. Regarding surgeon adherence to antibiotic prophylaxis guidelines, there was nonstatistical difference in the compliance rate to the hospital guidelines between different surgical specialties (*P* = 0.231) (Table 2).

A total of 90 (89%) surgical patients received antibiotic prophylaxis; of them 41 (40.6%) received appropriate antibiotic regimens and 49 (48.5%) patients received antibiotics other than those recommended by hospital guidelines. In the remaining 11 (10.9%) patients who did not receive surgical antibiotics prophylaxis during the study antibiotics were recommended but not given in 8 cases and antibiotics were not recommended and not given in 3 cases.

Table 3 shows the association between different types of antibiotic and their compliance with the hospital guidelines. Cefazolin (44.6%) was used most frequently in surgical prophylaxis. In 53.3% of procedures, The use of Cefazolin was in concordance with guidelines recommendations, while in 46.7% its use did not follow the guidelines of the hospital. The overall compliance of different types of antibiotics used according to the recommended guidelines showed statistically significant difference (*P* = 0.006) (Table 3).

## 4. Discussion

The current report analyzes the standard practice of care of surgeons at Hamad General Hospital regarding the compliance of SAP guidelines and the gaps in current practice to provide evidence for recommendations that may help to improve health care. The current study is unique in that it is the first time in our region to report the rate of compliance to SAP at a general hospital in a developing country. The use of SAP for minimizing the rate of SSI is effective and has been well established in the literature [14]. Based on the best available evidence to optimize the patient care and surgeon's practice, the American Society of Health System Pharmacists (ASHP) has developed therapeutic guidelines on antimicrobial prophylaxis in surgery [15]. Although such guidelines have been in place for many years, studies showed that inappropriate prophylaxis and poor adherence to guidelines are still major issues [16]. As in Table 4, several studies examining different surgical procedures showed a wide variation in the overall adherence to SAP guidelines, which ranges from 4.9% to 70.7% [17–24].

Therefore, it is important to assess and evaluate the current practice of SAP in a hospital to improve health care outcomes and reduce the gap between both practice and evidence based recommendations [25]. van Kasteren et al. [26] demonstrated that, in only 28% of cases, the overall adherence to all aspects of the guideline has been achieved. Earlier studies found a higher rate of noncompliance with respect to selection of appropriate antibiotics and recommended dosage, timing of administration, and duration of prophylaxis [24, 27–29]. In the present study, the compliance rate of antibiotic selection with the hospital infectious disease guidelines is 68.5%, while compliance rate of antibiotic duration with the hospital guidelines is 40.7%. Our findings are consistent with other studies which also evaluated the compliance with hospital SAP guidelines [24, 27, 28]. In 2011, a large study of 2373 patients in Tokyo found that the adherence rate for antibiotic selection was 53–84% while that adherence for antibiotic duration was 38–68% [30]. Interestingly, a French study of two-year duration separated by a 3-week period of targeted information showed that only 49% of prophylaxis was implemented appropriately before and after the given information. The authors concluded that the information program alone has no effect on the appropriate use of antibiotics for surgical prophylaxis [31]. Another retrospective study based on orthopedic trauma patients in Canada found that less than 32% of patients received recommended prophylaxis [32]. Further, a large surveillance study involving 8029 patients observed that only 35% of surgical antibiotic prophylaxis duration was appropriate [33].

The present report shows that cefazolin (44.6%) is the most frequently used antibiotic which corroborates with other studies in which cephalosporin antibiotics were the preferred choice in most of surgical procedures [34]. Also, our study shows that ceftriaxone is the third most common antibiotic used in surgical prophylaxis (17%). Broad spectrum antibiotics for surgical prophylaxis are recommended mainly for severe infection or in acute infection while waiting for the results of cultures [34].

In the present study, the compliance rate is significantly higher for clean-surgery as compared to clean contaminated surgery. Though, 43.6% of clean and 65.2% of the clean-contaminated procedures failed to demonstrate compliance with the recommendations. Mangram et al. [35] reported that particularly for clean surgery, although not recommended, surgeons preferred to give antibiotic prophylaxis. However, Tourmousoglou et al. [20] reported lower rate (19%) of noncompliance towards the adherence of general surgeons to national guidelines in patients undergoing clean surgery. Further, the investigators found that guideline adherence to appropriate antibiotic duration was comparable for clean (36%) and clean-contaminated (36.4%) surgeries. A recent prospective audit of SAP adherence in France found an overall compliance rate of 37%. In that study, the independent predictors of noncompliance to SAP guidelines included prescription of antimicrobial prophylaxis by a surgeon, clean-contaminated surgery, trauma-related surgery, and digestive tract, head and neck-related surgery [36].

The limitations of the current study include the involvement of small number of patients which did not give complete overview of the compliance rate among the different departments. The retrospective nature of the study is another limitation. Moreover, the current study did not analyze one important element of surgical antibiotic prophylaxis—the timing of antibiotic administration before incision. However, this is perhaps not crucial in the results as being noncompliant with one element of the prophylaxis is already considered as a guidelines deviation.

In conclusion, the effectiveness of SAP guidelines is well known; however, compliance with evidence based guidelines remains consistently poor. In our study, nonadherence was most commonly due to inappropriate choice of drug and use of antimicrobial prophylaxis for longer duration than recommended. This has the potential of ineffective prevention of SSI and emergence of resistant strains of bacteria within the institution. Deep evaluation of barriers that may hinder universal implementation of guidelines is warranted and solutions to increase adherence should be encouraged. It is evident from the literature that effective strategies which include addressing the knowledge and attitudes of staff together with quantitative and qualitative approaches help to improve the compliance rate with the SAP guidelines. Moreover, interactive workshops to address current controversies and solutions to overcome the compliance barrier are useful for enhancing surgical staff commitment towards hospital guidelines. Also auditing antibiotic use against agreed standards should be seen as a quality indicator to decrease the rate of SSI. The study highlights that there is a potential opportunity for a clinical pharmacist to facilitate evaluation of quality assured SAP management process across all surgical disciplines. Further, prospective studies are recommended to address these critical issues in more detail.

## Figures and Tables

**Table 1:** Overview of demographics and surgical antibiotic prophylaxis.

Number of patients (n = 101)	
Males (%)	80.2
Age (mean ± SD)	38.3 ± 16.9
Surgery class	
Clean (%)	54.5
Clean contaminated (%)	45.5
Antibiotics used (%)	89
Overall compliance	
Yes (%)	46.5
No (%)	53.5

**Table 2:** Assessment of surgeon adherence to antibiotic prophylaxis guidelines.

	Compliance	Noncompliance	*P* value
Surgery class			
Clean	31 (56.4)	24 (43.6)	0.024
Clean contaminated	16 (34.8)	30 (65.2)	
Surgery type			
Orthosurgery	15 (31.9)	13 (24.1)	0.231
GI surgery	18 (38.3)	18 (33.3)	
CABG	7 (14.9)	4 (7.4)	
OMF	0 (0)	4 (7.4)	
Surgery involves artificial device	2 (4.3)	4 (7.4)	
Neurosurgery	3 (6.4)	4 (7.4)	
Urologic surgery	2 (4.3)	7 (13)	

Results in parentheses are showing percentages.

**Table 3:** Association between different antibiotic types and compliance with hospital guidelines.

Antibiotic type	Total (*n* = 101)	Compliance (%)	Noncompliance (%)	*P* value
Cefazolin	45 (44.6%)	24 (53.3%)	21 (46.7%)	0.006
Cefuroxime	18 (17.8%)	13 (72.2%)	5 (27.8%)	
Ceftriaxone	17 (16.8%)	3 (17.6%)	14 (82.4%)	
Co-amoxicalv	6 (5.9%)	0 (0%)	6 (100%)	
Metronidazole	2 (1.9)	1 (50%)	1 (50%)	
Vancomycin	1 (0.9%)	0 (0%)	1 (100%)	
Ciprofloxacin	1 (0.9%)	0 (0%)	1 (100%)	

**Table 4:** 

Country	Study duration (months)	Overall compliance rate (%) of SAP guidelines	Reason for noncompliance with SAP guidelines			
			Inappropriate antibiotic		Inappropriate antibiotic selection (%)	Inappropriate administration of indicated SAP (%)
			Duration (%)	Time of administration for 1st dose (%)		
Brazil [17]	5	4.9	95.2	15.3	19.1	98.1
Australia [18]	33	—	12.4	7.1	1.7%	—
Italy [19]	1	44.8	—	24.3	—	17.3
Greece [20]	10	—	63.7	0.0	30	19
Jordan [21]	3	—	60.6	0.9	98.3	0
India [22]	—	—		87	—	19
Eritrea [23]	3	—	—	—	—	34
Germany [24]	6	70.7	32.9	—	—	—
Present study	3	46.5	59.3	—	31.5	9.2

SAP: surgical antibiotic prophylaxis.
